# A confirmatory factor analysis of the knowledge, attitude and practice questionnaire towards prevention of respiratory tract infections during Hajj and Umrah

**DOI:** 10.1186/s12889-020-09756-5

**Published:** 2020-11-10

**Authors:** Mohammed Dauda Goni, Nyi Nyi Naing, Habsah Hasan, Nadiah Wan-Arfah, Zakuan Zainy Deris, Wan Nor Arifin, Aisha Abubakar Baaba, Stanley Njaka

**Affiliations:** 1 Ministry of Agriculture and Natural Resources, IBB Secretariat Complex, Yobe State Government, Damaturu, Nigeria; 2 Faculty of Medicine, Medical Campus Universiti Sultan Zainal Abidin, 20400 Kuala Terengganu, Malaysia; 3 Department of Microbiology and Parasitology, School of Medical Sciences, Universiti Sains Malaysia, 16150 Kubang Kerian, Kelantan Malaysia; 4 Faculty of Health Sciences, Gong Badak Campus, Universiti Sultan Zainal Abidin, 21300 Kuala Nerus, Terengganu Malaysia; 5 Unit of Biostatistics and Research Methodology, School of Medical Sciences, Universiti Sains Malaysia Health Campus, 16150 Kubang Kerian, Kelantan Malaysia; 6 Centre for Language Studies and Generic Development, Universiti Malaysia Kelantan, Locked Bag 01, 16300 Bachok, Kelantan Malaysia; 7 Department of Nursing Science, School of Health Sciences, Universiti Sains Malaysia Health Campus, 16150 Kubang Kerian, Malaysia

**Keywords:** Respiratory tract infection, Confirmatory factor analysis, Knowledge, Attitude, Practice

## Abstract

**Background:**

Respiratory tract infections are one of the common infection associated with Hajj pilgrimage that is of great public health and global concern. This study is aimed at determining the factor structure of the knowledge, attitude, and practice questionnaire for the prevention of respiratory tract infections during Hajj by confirmatory factor analysis (CFA).

**Methods:**

A multistage cluster sampling method was conducted on Malaysian Umrah pilgrims during the weekly Umrah orientation course. A total of 200 Umrah pilgrims participated in the study. The knowledge, attitude and practice (KAP) questionnaire was distributed to pilgrims at the beginning of the orientation and retrieved immediately at the end of the orientation. Data analysis was done using R version 3.5.0 after data entry into SPSS 24. The robust maximum likelihood was used for the estimation due to the multivariate normality assumption violation. A two-factor model was tested for measurement model validity and construct validity for each of the attitude and practice domains.

**Results:**

CFA of a 25-item in total, the two-factor model yielded adequate goodness-of-fit values. The measurement model also showed good convergent and discriminant validity after model re-specification. A two-factor model was tested for measurement model validity and construct validity for each of the attitude and practice domains. The result also showed a statistically significant value (*p* < 0.001) with χ^2^ (df) values of 76.8 (43) and 121 (76) for attitude and practice domains, respectively.

**Conclusion:**

The KAP questionnaire was proven to have a valid measurement model and reliable constructs. It was deemed suitable for use to measure the KAP of Hajj and Umrah pilgrims towards the prevention for all respiratory tract infections.

## Background

The Holy pilgrimage to Mecca, Saudi Arabia is one of the five cardinal pillars of worship upon every financially and physically able Muslim individual. Hajj is among the largest mass gathering in the world, with approximately 2 million pilgrims participating from different countries every year [1]. This poses a great risk to public health considering the overcrowding, presence of comorbidities among the pilgrims and adverse climatic condition are huge challenges to both participating and the host countries especially regarding infectious diseases such as respiratory tract infections [2]. On the other hand, Umrah also known as Lesser Hajj can be performed at any time of the year and is not obligatory on Muslims; however, is a highly significant religious practice [3].

Respiratory tract infections are the most prevalent illnesses spread throughout the Hajj period, and influenza virus and rhinovirus are the most commonly reported respiratory viruses among pilgrims [4]. However, a high prevalence of respiratory tract illnesses is still reported among Malaysian Hajj pilgrims at over 90% despite the implementation of different preventive measures over recent years [5].

The current pandemic due to the novel severe acute respiratory syndrome coronavirus 2 (SARS-CoV-2) which was first reported in Hubei province of China in December 2019 has prompted many researchers with the development of a valid and reliable tool for the measurement its knowledge, attitude and practice (KAP) among various communities [6]. The World Health Organization (WHO) declared the infection as a pandemic in March 2020 [7] with more than 9 million confirmed cases and 469,239 deaths due to of Covid-19 from 188 countries across the world based on the figures from the Johns Hopkins University Coronavirus resource centre [8]. Similarly, Saudi Arabia, being the sole host of the Hajj and Umrah pilgrimage, has recorded 161,005 confirmed cases of COVID-19 resulting in more than 1300 deaths [8]. This has prompted Saudi Arabia authorities to initiate more proactive protective precautions such as temporary suspension of Umrah pilgrimage and limiting Hajj 2020 pilgrimage to only a few Saudi residents with strict guidelines on the rules of social distancing, the use of face mask and proper hand hygiene [9–11].

Confirmatory factor analysis (CFA) is advance construct validity and superior to exploratory factor analysis (EFA) and simple reliability analysis (test-retest and internal consistency reliabilities) in several ways. CFA is also a kind of structural equation modelling (SEM) that is related to measurement models [12]. The application of CFA is worthwhile to support the links connecting items and their respective domains. This permits the fixing of these relations in the measurement model and presents measures to evaluate the fit of the proposed theoretical model to the collected data [13]. Therefore, CFA is regarded as a vital means for validation in the social and behavioural sciences [12]. Measurement scale development involves numerous processes and protocols to establish its validity and reliability. The content and characteristics of the basic constructs and the choice of items to be included can also be established in a pilot study or adopted from a previous similar study and validated by CFA [14]. The application of improper measurement tools that are not validated can lead to inaccurate and misleading findings, resulting in a poor plan for interventions and therefore, too unreliable efficacy [15]. The Item Response Theory models (Rasch model) utilizes the principle of true Score models which comprises a collection of dogmatic formulae for systematic analysis to achieve the desired objective [16].

So far, few studies specifically reported the knowledge, attitude and practice of various respiratory tract infections preventive behaviours by Hajj pilgrims [17–23], however, none of these studies were documented to have employed a questionnaire that is properly developed and validated. Therefore, this study was aimed at determining the construct validity and reliability of the knowledge, attitude and practice (KAP) questionnaire towards the prevention of respiratory tract infections during Hajj and Umrah among Malaysian Hajj pilgrims.

## Methods

### Research design and study population

A cross-sectional study was carried out among Malaysian Umrah pilgrims attending a weekly Umrah orientation course organized by private Umrah tour companies from March to June 2018. This study was the second stage of a large study [24–26]. In the first stage, we conducted an exploratory factor analysis of the measurement tool [24].

### Sample size and sampling method

A total of 200 Umrah pilgrims were recruited through a multistage sampling method for the 72 items in the KAP questionnaire for prevention of respiratory tract infection (RTI) during Hajj. The sample size for this study was based on a simulation study, as recommended by Hair et al. (2010) [27] for CFA. Therefore the sample size for this study was fixed at *n* = 200 when the anticipated domains were seven or less, and items commonality was less than 0.5 and no under identified domains.

The sampling method used was done in two stages. The first stage was a purposive selection of private Hajj and Umrah companies as clusters. Hajj/Umrah travel companies were eligible if: 1) they were located in Kota Bharu, Kelantan; 2) they conduct weekly Hajj/Umrah orientation courses, and if; 3) the management was willing to participate actively in the study and to collaborate with the researcher from Universiti Sains Malaysia. Five Hajj/Umrah tour companies were identified and contacted about the project, and only two companies agreed to participate. The participants were approached during the routine orientation courses conducted by the Hajj/Umrah travel companies for the pilgrims after being briefed about the validation study and seeking consent from them by research assistants in Malay language by three (3) research assistants. The questionnaires were distributed to the pilgrims at the beginning of the course and collected back at the end of the day’s session. Incentives were provided to the participants.

### Measurement tool

A self-administered questionnaire for the measurement of pilgrim’s KAP towards the prevention of RTIs as used in a previous study [24] was used in this study. All the domains, as well as the sub-domains, have been developed and exploratory factor analysis (EFA) was done [24].

### Data collection procedures

All data were collected from June 2018 to August 2018. A self-administered questionnaires were distributed to the Umrah pilgrims before their weekly course that met the inclusion criteria. Pilgrims that are aged 18 years and above, able to write and speak in Bahasa Malay and are willing to participate are considered to have fulfilled the inclusion criteria. Participants were briefed on the purpose of the study, the procedures, and the confidentiality of their responses. Informed consent was obtained from the participants that are willing to be part of the study prior to the administration of the questionnaire. The pilgrims were also instructed to give their honest responses when answering the questionnaire. The completed questionnaire was immediately retrieved from the participants at the end of the day’s orientation. The time to complete the questionnaire was approximately 10 to 15 min.

### Data management and preliminary analysis

All data were entered and checked for missing data using SPSS software version 24 and then transferred to R version 3.5.0 for Item Response Theory (IRT) and Confirmatory factor analysis (CFA) analysis. Data analysis was done using R version 3.5.0 in the R Studio environment.

### Item response theory (IRT)

Considering the dichotomous outcome of the responses of the items in the knowledge section, two-parameter logistic item response theory (2-PL IRT) analysis was done using the ltm package version 1.0.0 6.

### Confirmatory factor analysis (CFA)

Confirmatory factor analysis (CFA) was conducted to confirm the factorial structure of the KAP questionnaire identified in the EFA published in the other part of this study. The attitude and practice domains were analysed as recommended by lavaan package version 0.5–22 [28]. Several indices indicated a good model fit for the construct, they include: the ratio of chi-square to degree of freedom (*χ*^2^/df) < 5.0, root mean square error of approximation (RMSEA) ≤0.08, comparative fit index (CFI) > 0.9, Tucker Lewis Index (TLI) > 0.9, and *p* > 0.05 for the chi-square test [29]. For composite reliability, semTools package version 0.4–14 5–6 was used to determine the Raykov’s rho [29, 30]. Hair et al. [27] suggested that model fitness can be decided by at least a minimum of three different indices. A good relationship between items and respective factors are shown by a standardized factor loading greater than 0.5 as well as a *p*-value of less than 0.05 and it therefore further proves the validity of the construct. Composite reliability of the domains was calculated with a value of 0.7 and above was considered acceptable [27, 31].

## Results

A total of 200 Umrah pilgrims responded to this study. On data screening, no missing data was found. The age of the participants from this study ranged from 18 to 80 years old with, a mean age of 39.13 (SD 16.03). The females (65.5%) dominated the number of pilgrims. The socio-demographic characteristics of the participants are shown in Table 1.

**Table 1:** Socio-demographic characteristics of participants (*n* = 200)

Variables	Mean (SD)	Frequency (%)
Age (years)	39.13 (16.029)	
Gender		
Male		65 (35.5)
Female		131 (65.5)
Ethnicity		
Malay		197 (98.5)
Indian		1 (0.5)
Others		2 (1.0)
Marital status		
Single		89 (44.5)
Married		109 (54.5)
Divorced/widowed		2 (1.0)
Occupation		
Student		19 (9.5)
Civil servant		37 (18.5)
Private sector		95 (47.5)
Pensioner		22 (11)
Housewife		15 (7.5)
Self-employed		12 (6)
Highest level of education		
PhD		4 (2.0)
Master’s degree		13 (6.5)
Bachelor’s degree		42 (21.0)
Diploma		73 (36.5)
Secondary school		54 (27.0)
Primary school		14 (7.0)
History of vaccination		
Meningococcal vaccine		60 (30)
Influenza (flu) vaccine		29 (14.5)
Pneumococcal vaccine		24 (12.0)
Presence of Co-morbidities		
Chronic lung disease		1 (0.5)
Neuromuscular disease		9 (4.5)
Allergic rhinitis		2 (1.0)
Diabetes		6 (3.0)
Hypertension		29 (14.5)
Heart disease		2 (1.0)
Chronic kidney disease		2 (2)
Immune deficiency disorders		1 (0.5)

In the knowledge section, IRT analysis results showed an acceptable range for both difficulty (− 3 to + 3) and the discrimination parameter on each of the items in all the sub domains. The sub-domains are SD1 (K1i, K1ii, K1iii), SD2 (K2i, K2ii, K2iii, K2iv, K2v, K3), SD3 (K4i, K4ii, K4iii, K4iv, K4v and K4vi), SD4 (K5i, K5ii, K5iii, K5iv and K5v), prevention practices (K6i, K6ii, K6iii, K6iv and K6v) and SD5 (K7i, K7ii, K7iii, K8 and K9) covering the aetiology, transmission, risk factors, complications, preventive practices and the use of personal protective equipment (PPE). However, all the items were retained because they had acceptable difficulty and discrimination values. The amount of information tapped by the items between − 3 and + 3 difficulty range was 93.1%. The unidimensionality assumption was not supported by the modified parallel test at α = 0.05 (*p* = 0.010). In terms of internal consistency reliability, Cronbach’s alpha was 0.9. IRT analysis for the psychometric characteristics of the domain, as shown in Table 2.

**Table 2:** Result of the IRT analysis in the knowledge section (*n* = 200)

Items	*b*	*a*	λ	χ^2^ (df = 8)	*P* values
1 Flu-like illnesses are caused by:					
i Allergies	−0.41	3.50	0.9	100.55	**< 0.001**
ii Bacteria	−0.54	2.16	0.78	62.98	**< 0.001**
iii Virus	−1.04	3.12	0.87	120.2	**< 0.001**
2 Flu-like illnesses are spread by:					
i Air	−1.10	3.39	0.88	24.34	**0.002**
ii Dust	−0.86	2.24	0.79	26.19	**0.001**
iii Sharing towels with an infected person	−0.32	3.95	0.94	52.78	**< 0.001**
iv Water	0.24	2.32	0.82	88.76	**< 0.001**
v Shaking the hands of an infected person with a cough and/or cold	−0.16	1.90	0.75	52.42	**< 0.001**
3 Flu-like illnesses are spread quickly	−1.17	1.41	0.64	101.32	**< 0.001**
4 The following persons are at an increased risk of flu-like illnesses:					
i Asthmatics	−0.87	2.83	0.86	43.63	**< 0.001**
ii Diabetics	0.40	4.32	0.93	21.16	**0.007**
iii People with arthritis	0.43	2.34	0.80	50.84	**< 0.001**
iv Senior citizens aged 65 and older	−0.57	1.94	0.75	29.26	**< 0.001**
v Smokers	−0.14	3.08	0.87	75.46	**< 0.001**
vi Those in crowded places/among a lot of people	−1.13	1.83	0.73	49.71	**< 0.001**
5 What are the complications of flu-like illnesses?					
i Bronchitis	−0.14	2.93	0.91	126.49	**< 0.001**
ii Difficulty in breathing	0.64	6.26	0.88	22.80	**0.004**
iii Multi-organ failure	0.55	2.85	0.89	170.57	**< 0.001**
iv Pneumonia	−0.27	2.76	0.90	91.12	**< 0.001**
6 The following practices can help protect you from flu-like illnesses:					
i Covering your nose with your hands	−0.67	1.75	0.92	71.99	**< 0.001**
ii Ensuring a healthy diet	−1.05	2.29	0.50	38.14	**< 0.001**
ii Receiving vaccinations	−0.80	2.26	0.85	66.51	**< 0.001**
iv Washing your hands with hand sanitizers	−0.86	6.29	0.78	15.08	**< 0.001**
v Wearing a face mask	−1.22	5.07	0.71	10.75	**< 0.001**
7 The following are reasons for wearing a mask:					
i Being in crowded places	−1.03	6.11	0.97	11.82	**< 0.001**
ii Being near people who are coughing	−1.26	4.83	0.96	20.75	**0.008**
iii When I am sick	−0.91	4.33	0.94	49.03	**< 0.001**
8 A cloth facial mask is as effective as a 2-ply surgical facial mask	1.02	1.29	0.60	60.85	**< 0.001**
9 If I am not sick, the used face mask can be stored in a bag for later use	0.72	1.56	0.67	182.10	**< 0.001**

For the attitude domain, the two-factor model was then tested by CFA using an MLR estimation method. MLR was used because the data did not follow a multivariate normal distribution required by the MLR. Satisfactory model fitness was not demonstrated by the initial 12-item factor. To achieve the model fitness, the maximum likelihood (ML) values were examined and re-analysed to achieve a better model fit. To be included in the model, items with high correlated errors within the same factor will be considered. The two-factor model showed a good fit (χ^2^ [df = 6] = 43, *p* < 0.001; CFI_robust_ = 0.928; TLI_robust_ = 0.890; RMSEA_robust_ = 0.063; SRMR = 0.079) as shown in Table after correlated errors (A12A↔A13, *r* = 0.341; A3↔A9, *r* = − 0.267; A5A↔A5B, *r* = 0.265; A8↔A7, *r* = 0.268; A8↔A9, *r* = 0.240; A10↔A4, *r* = − 0.237; A10↔A7, *r* = − 0.191; A3↔A5B, *r* = 0.267; A9↔A5B, *r* = − 0.168; A10↔A5B, *r* = 0.205) were added. However, the two sub-domains under attitude (barriers to compliance and self-motivation) have a correlation between them of *r* = 0.444. The composite reliability of the barriers to compliance and self-motivation factor all have a satisfactory cut-off value of > 0.7 as summarize in Table 3.

**Table 3:** Results of CFA of the attitude section

Factors	Items	Factor loading	Reliability (Raykov’s rho)
Barriers to compliance	A3: Since the bird flu, SARS, MERS-COV and H1N1 crises are over, I no longer need to worry about contracting flu-like illnesses	0.696	0.76
	A8: I am generally opposed to wearing a face mask	0.555	
	A9: Flu vaccinations have unpleasant side effects	0.376	
	A10: I am influence by negative news about flu vaccines	0.751	
	A11: It is too much trouble to get a flu vaccine	0.751	
Self-motivation	A4 If I have a flu-like illness, I may spread it to others	0.516	0.72
	A5: I feel that someone that have influenza-like illness should:		
	A5A: cover his mouth and nose with his bare hand when coughing or sneezing	0.603	
	A5B: cover his mouth and nose with a handkerchief when coughing or sneezing	0.402	
	A6: Influenza vaccines protects hajj pilgrims from influenza	0.75	
	A7: Using a hand wash can prevent you from getting flu like illness	0.652	
	A12A: I think coughs and the flu can be prevented by wearing a mask outside my house	0.424	
	A13: Wearing a well-fitting face mask is effective in preventing flu-like illnesses	0.431	

For practice domain which comprises of 13 items, the two-factor model was analyzed by CFA. The model showed an acceptable fitness, as shown in Table 4 (χ^2^ [df = 64] = 31.49, *p* < 0.001; CFIrobust = 0.903; TLIrobust = 0.882; RMSEArobust = 0.073; SRMR = 0.067). The correlations between the factors were: Healthy-lifestyle↔Prevention-practices (r = 0.471). The composite reliability of the healthy lifestyle and prevention practices factors were above the cut-off value of 0.7 (Raykov’s rho = 0.863 and 0.827), despite the low standardized loading for item P7.

**Table 4:** Results of CFA of the practice domain

Factors	Items	Factor loading	Reliability (Raykov’s rho)
Health lifestyle	P1: I eat vegetables	0.918	0.863
	P2: I eat fruits	0.888	
	P5: I use soap to wash my hands	0.664	
Prevention practices	P4: When wearing a mask, I test it to ensure it fits properly	0.535	0.827
	P6: I use disinfectant or disposable wipes or hand gel to wash my hands	0.483	
	P7: I use a washable cloth handkerchief to clean my hands	0.284	
	P8: I wash my hands after:		
	P8A: touching the personal items of someone who has a cough and/or cold	0.744	
	P8B: shaking hands with people who have a cough and/or cold	0.787	
	P8C: touching doorknobs	0.692	
	P9: I refrain from:		
	P9A: being close to those who cough or sneeze	0.562	
	P9B: shaking the hands of those who have a cough and/or cold	0.577	
	P9C: often touching my nose	0.365	
	P10: I received the flu vaccine	0.511	

The model showed an acceptable fitness for both attitude and practice. In the attitude domain, two-factor model showed a good fit (χ^2^ [df = 6] = 43, *p* < 0.001; CFI_robust_ = 0.928; TLI_robust_ = 0.890; RMSEA_robust_ = 0.063; SRMR = 0.079) after correlated errors (A12A↔A13, *r* = 0.341; A3↔A9, *r* = − 0.267; A5A↔A5B, *r* = 0.265; A8↔A7, *r* = 0.268; A8↔A9, *r* = 0.240; A10↔A4, *r* = − 0.237; A10↔A7, *r* = − 0.191; A3↔A5B, *r* = 0.267; A9↔A5B, *r* = − 0.168; A10↔A5B, *r* = 0.205) were added. For the practice domain, the fitness indices (χ^2^ [df = 64] = 31.49, *p* < 0.001; CFIrobust = 0.903; TLIrobust = 0.882; RMSEArobust = 0.073; SRMR = 0.067) are well represented. The fitness indices are summarized in Table 5.

**Table 5:** Fit Indices for Confirmatory Factor Models

Factors	No of items			Goodness of fit indices				
		*X*^*2*^(df)	P value	df	CFI	TLI	RMSEA	SRMR
Attitude	12	76.8 (43)	< 0.001	66	0.928	0.890	0.063	0.079
Practice	13	121 (76)	< 0.001	64	0.903	0.882	0.073	0.067

## Discussion

This study validated a Malay questionnaire for the KAP evaluation of Hajj pilgrims towards the prevention of respiratory tract infections. Overall, the results of the CFA for all the domains indicated that the measurement models for each construct are fit except the attitude domain that undergone through a model modification to improve the model fit. Finding from this study could not be compared with the psychometric properties from other studies conducted on the knowledge, attitudes and practices on respiratory tract infection due to the paucity of documented and described the validation process. Our findings support the originally developed two-factor sub-domain for each of the attitude and practice.

Based on the assumptions checking for multivariate, the data were not normally distributed for CFA. Therefore, MLR was the preferred method for fitting the CFA model to turn over the violation of the normality of the multivariate analysis. Due to the aforementioned reason, estimation of MLR was done using robust (Huber-White) with standard errors and a scaled test statistic that is hypothetically matched the Yuan-Bentler test statistic [31–33].

Our findings showed a reasonably good fit for the questionnaire, giving confirmatory details for the factor structure for both domains. All the fit indices (RMSEA, CFI, TLI, SRMR) are within acceptable values and therefore supported the construct validity [34]. There are numerous studies done in Malaysia which support the accepted values of the fit indices, which is similar to the present study results [35–37].

The reliability of the various domains was based on the Raykov’s rho which accounts for what of each individual item stands for and its latent error; however, they provide much less biased estimate of Cronbach’s alpha. The attitude and practice factors of the KAP questionnaire had good reliability, as shown by the reliability coefficients exceeding 0.70.

In this study, like much other research, has some limitations. Firstly, data were collected from Umrah pilgrims using a sampling that is non-random in nature and thus should not necessarily be considered representative of the population and may not be a similar experience to Hajj pilgrimage. Secondly, majority of the participants are of Malay race, future research should incorporate other race to make it heterogenous population. Finally, as stated earlier, this is the first study on confirmatory factor analysis of KAP on respiratory tract infection prevention in Malaysia and therefore comparison to other studies is not possible.

## Conclusions

The KAP questionnaire has shown to have good validity, reliability and psychometric properties towards measuring knowledge, attitude and practice of Malaysian Hajj pilgrims towards prevention of respiratory tract infection. This article could serve as a template for the implementation of various studies in community settings amidst the current Covid19 pandemic for effective prevention and control strategies.

## Data Availability

The datasets used and/or analysed during the current study are available from the corresponding author on reasonable request.

## Abbreviations

**RTI**: Respiratory tract infection
**IRT**: Item response theory
**CFA**: Confirmatory factor analysis
**KAP**: Knowledge, attitude, and practice
